# A Case of Pelvic Abscess Caused by *Edwardsiella tarda* followed by Laparoscopic Resection of a Hematoma Derived from Caesarean Section

**DOI:** 10.1155/2018/4970854

**Published:** 2018-05-22

**Authors:** Koji Yamanoi, Koji Yasumoto, Jumpei Ogura, Takahiro Hirayama, Koh Suginami

**Affiliations:** ^1^Department of Obstetrics and Gynecology, Toyooka Public Hospital, Toyooka, Japan; ^2^Department of Dermatology, Cutaneous Biology Research Center, Massachusetts General Hospital, Boston, MA, USA

## Abstract

*Edwardsiella tarda* (*E. tarda*) infections are rare and can be fatal. We report a case of an *E. tarda* abscess which developed in the hematoma originally derived from a caesarean section. A 24-year-old gravida 1 woman was admitted to our hospital with a complaint of abdominal pain. Approximately one month before her admission, pelvic hematoma had developed derived from caesarean section. Followed by the failure of conservative management, she underwent laparoscopic surgery to remove the hematoma 6 days before her admission. On computed tomography examination, we found that the abscess with a diameter of 9 cm was located in the right pelvic space. We punctured the abscess and identified *E. tarda* in the abscess. We continued administering antibiotics, but her symptoms, including fever and abdominal pain, became worse, and the abscess enlarged. We performed laparotomy drainage and ileocecal resection on the 10th posthospitalization day. After drainage surgery, the patient's condition improved gradually, and the patient was discharged uneventfully. There are no reports in patients of *E. tarda* infection during the perinatal period. *E. tarda* infection can be a life-threatening illness even in immunocompetent patients. In the case of *E. tarda* infection, intensive care and surgical procedures should be considered.

## 1. Introduction

*Edwardsiella tarda* (*E. tarda*) is a member of the family Enterobacteriaceae and, along with *E. ictaluri* and *E. hoshinae*, constitutes the genus *Edwardsiella* [1]. It is a Gram-negative, motile, facultatively anaerobic rod-shaped bacterium that is most commonly isolated from fresh-water marine organisms [2].

The most common disease caused by *E. tarda* infection in humans is gastroenteritis [3]. Extraintestinal *E. tarda* infections are rare and can be fatal. It has been reported that extraintestinal *E. tarda* infections occur in compromised hosts, such as patients with cancer, diabetes mellitus, and an immunosuppression status [4, 5]. Furthermore, to our knowledge, there are no reports of the occurrence of extraintestinal *E. tarda* infection in noncompromised hosts, particularly pregnant and postpartum patients.

Here, we report a case of a pelvic abscess caused by *E. tarda* infection which developed in the hematoma originally derived from a cesarean section.

## 2. Case

### 2.1. Emergency Cesarean Section and Development of a Hematoma

The patient was a 24-year-old woman, 1 gravida and 0 para. She was diagnosed with a normal intrauterine pregnancy. There was no abnormality in her medical history and allergy history.

At 36 weeks and 2 days of gestation, she was diagnosed with a placental abruption and underwent emergency cesarean section immediately. A female baby weighing 2439 g was delivered with umbilical artery pH of 7.140. The amount of bleeding during surgery was approximately 658 ml including the amniotic fluid. Until the 2nd postoperative day, there were no abnormalities in her vital signs or urinary volume.

**Figure 1:**
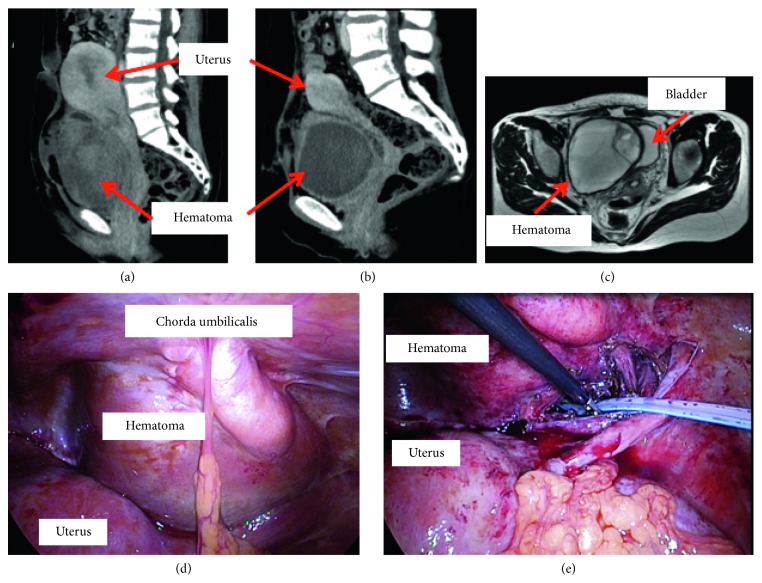
Occurrence of hematoma after caesarean section and laparoscopic surgery to treat it. (a) Contrast CT image taken on the 3rd postoperative day. Hematoma of 9 cm in diameter existed at the isthmus of uterus. Red arrow indicates hematoma and uterus. (b) Contrast CT image taken on the 28th postoperative day. Hematoma of same size remained in the same place. Red arrow indicates hematoma and uterus. (c) MRI (T2-weighted image) findings of hematoma taken on the 28th postoperative day. Encapsulated hematoma existed. Red arrow indicates hematoma and bladder. (d) Intraoperative findings. Hematoma existed extraperitoneally, inside chorda umbilicalis. (e) We cut Cutting of peritoneum to reach inside of hematoma. Drainage tube was set.

Blood tests were performed on the 3rd postoperative day and revealed severe anemia with a hemoglobin (Hb) level of 3.9 g/dl. Performing an abdominal ultrasound examination and enhanced computed tomography (CT) of the lower abdomen, we found a hematoma with a diameter of 9 cm between the uterus and bladder (Figure 1). A blood transfusion improved the anemia immediately. She did not complain of abdominal discomfort, and no signs of infection were observed. Thus, a conservative therapy was chosen. She was discharged on the 10th postoperative day and was followed as an outpatient.

On the 28th postoperative day, she came to our department with a complaint of abnormal vaginal bleeding. Blood tests did not reveal anemia (Hb 12.0 g/dl). Enhanced CT and magnetic resonance imaging (MRI) were performed, and there were no apparent findings of infection associated with the hematoma; however, we were concerned about a fistula at the site of the uterine incision. To remove the hematoma and check the incision site, we performed laparoscopic surgery.

We inserted a 12 mm port in the umbilical region and observed the abdominal cavity. The hematoma was located extraperitoneally inside the chorda umbilicalis (Figure 1). Then, we performed resection of the hematoma laparoscopically. We cut the peritoneum inside the chorda umbilicalis and successfully reached the interior of the hematoma. The uterine incision site was located away from the hematoma; therefore, we thought that the fistula was not located between the incision and hematoma. We resected the hematoma and lavaged the space fully, followed by the placement of a drainage tube.

The patient had an uneventful postoperative course, and the drainage amount was small. We removed the drainage tube on the 4th postoperative day, and the patient was discharged on the same day.

Two days after her discharge, the patient returned to the emergency department of our hospital with a complaint of severe diarrhea, gastric discomfort, and a high fever.

### 2.2. The Course of Treatment after Emergent Admission

**Figure 2:**
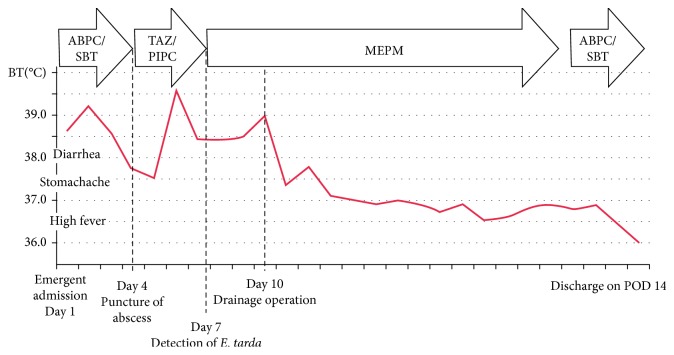
Treatment process and change of body temperature after emergent admission.

The change in the patient's body temperature and treatment process is shown in Figure 2.

**Figure 3:**
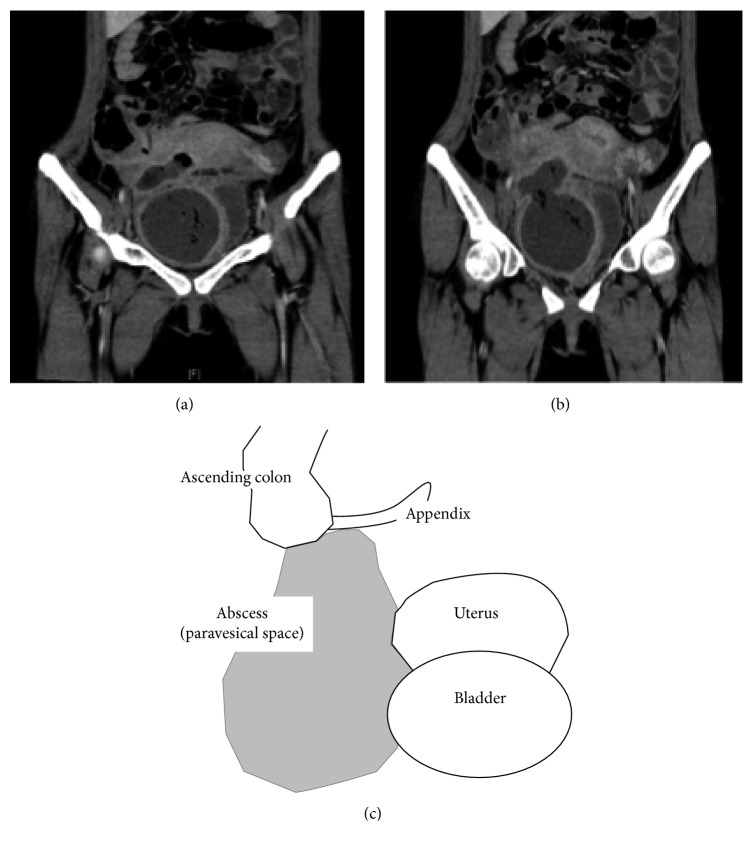
Imaging findings in the treatment of abscess and abscess position. (a) Enhanced CT image taken at the day of emergent admission. The density of abscess was relatively high, and air was detected. (b) Enhanced CT image taken at the 8th postadmission day. Abscess got large and reached to serosa of ileum. (c) Location of abscess is shown.

At admission, the patient complained of a high fever, severe diarrhea, and intense abdominal pain. Enhanced CT was performed and revealed that a cyst with a diameter of 9 cm was located at the same site where the hematoma had existed (Figure 3(a)). The CT value inside the cyst was slightly elevated. Blood tests were performed, and the results showed a slight elevation of the WBC count and C-reactive protein (CRP) (10,800/*µ*l and 1.6 mg/dl, resp.). We assumed that an infection occurred at the site of the hematoma, which resulted in the abscess. We administered antibiotics, namely, ABPC/SBT.

The antibiotics improved the diarrhea immediately; however, her body temperature was in the latter half of the 37°C range. Abdominal pain was improved to some degree. On the 4th postadmission day, we punctured the abscess transvaginally and absorbed dirty gray pus. We considered an anaerobic bacterial infection and changed the antibiotic from ABPC/SBT to TAZ/PIPC.

On the 7th postadmission day, her body temperature exceeded 38°C and her gastric discomfort tended to worsen. On the same day, bacterial culture examination of the pus revealed that *E. tarda* was detected. We performed the literature survey and found that abscess of *E. tarda* could be fatal. The patient's general condition tended to worsen. Although the antibiotics sensitivity test revealed that *E. tarda* was susceptible to almost all antibiotics, including ABPC/SBT and TAZ/PIPC, we thought that escalation of antibiotics was preferred. Consulting with the infection care team, we changed the antibiotics from TAZ/PIPC to MEPM.

Her fever still remained above 38°C (Figure 2), and her gastric discomfort tended to worsen each day. On the 9th postadmission day, we performed an enhanced CT scan and found an increase in abscess size (Figure 3(b)), which expanded into the peritoneal cavity, close to the ileocecal region. We decided that a drainage procedure was necessary.

On the 10th postadmission day, we performed a laparotomy to facilitate the drainage procedure. Approximately 200 ml of dark gray pus with a bad odor was absorbed. The abscess was mainly located in the obturator space surrounded by the bladder, uterus, ileum, and external iliac vein (Figure 3(c)). We resected the infected granulation tissue attached to the surface of the uterus, bladder, and pelvic wall and washed the area very carefully. The cranial side of the abscess expanded into the abdominal cavity and serosa of the ileum. The serosa of the ileum had become fragile. Although no apparent findings of perforation were observed, we resected the ileum to prevent the subsequent occurrence of perforation or diverticulum. Then, we placed a drainage tube and finished the procedure.

*E. tarda* was detected from the drainage by bacterial culture examination. After the operation, her body temperature tended to decrease to below 37°C (Figure 2). The WBC count and CRP value tended to decrease to 6300/*µ*l and 0.61 mg/dl, respectively, on the 7th postoperative day. On the same day, we changed antibiotics from MEPM to ABPC/SBT. We removed the drainage tube on the 17th postoperative day. The antibiotic regimen was completed, and the patient was discharged on the 21st postoperative day.

**Figure 4:**
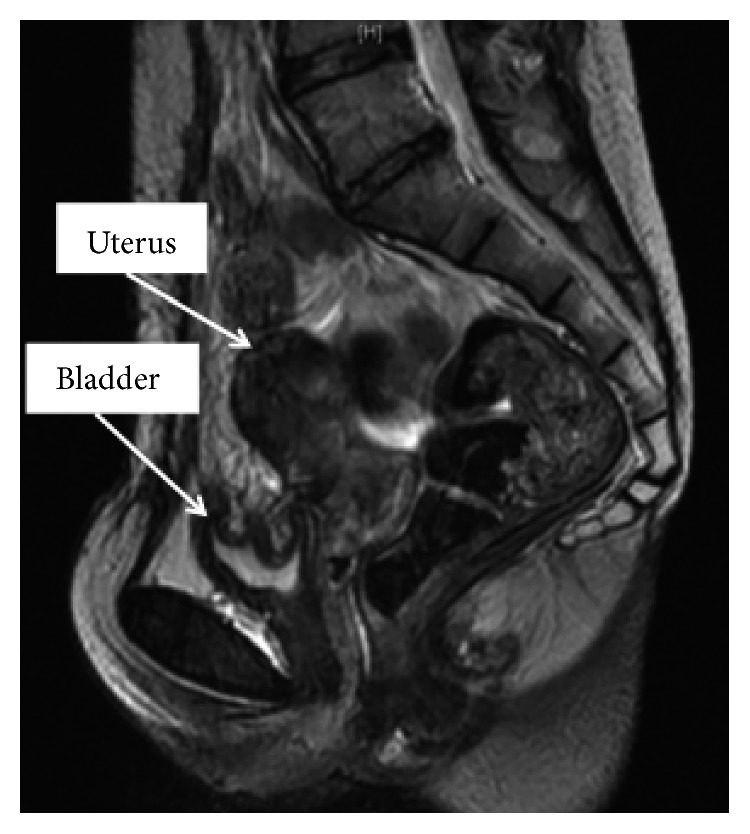
MRI image taken 4 months after drainage operation. Abscess or cyst could not be detected.

An adhesion ileus occurred on the 30th postoperative day, but it improved using conservative therapy. Four months after the operation, we performed a pelvic MRI examination and found no abnormal findings in her uterus, bladder, and pelvic space (Figure 4).

The patient is currently more than one and a half year post-op and has reported no complaints.

## 3. Materials and Methods for Detecting *E. tarda* and Antibiotic Sensitivity Test

The specimen was cultured aerobically onto Nissui Plate Sheep Blood Agar (Nissui Pharmaceutical, Tokyo, Japan) and Nissui Plate DHL Agar (Nissui Pharmaceutical) at 35°C for 18 hours. For detection of *E. tarda* and antibiotic sensitivity test of *E. tarda*, VITEK® GN detection card and sensitivity card (AST-N28) (bioMerieux Japan, Tokyo, Japan) were used.

## 4. Discussion

*E. tarda* is known to live in water and is detected in fish, amphibians, and reptiles. It has been reported that ingesting fish, food, or water can cause infection of *E. tarda* in humans [3]. The most common manifestation is enteritis, which can be characterized by symptoms that include vomiting and diarrhea. Gastrointestinal disease caused by *E. tarda* is more common in tropical and subtropical areas [3].

An *E. tarda* extraintestinal infection is relatively uncommon. The major underlying factor that predisposes individuals to *E. tarda* extraintestinal infections is compromised hosts, such as those with malignancy and diabetes mellitus. Taguchi et al. reported an *E. tarda* abscess in a patient with diabetes mellitus [4]. Mizunoe et al. reported an *E. tarda* abscess in a patient with malignancy [5]. Ingestion of a raw fish or exposure to an aquatic environment is also associated with increased risk of *E. tarda* extraintestinal infection [6]. Colub et al. reported an *E. tarda* tuboovarian abscess in a patient who had a history of raw fish ingestion [7]. As for cases of *E. tarda* extraintestinal infections in patients during the perinatal or postnatal period, Mikamo et al. reported a case of *E. tarda* endometritis that occurred in the puerperium in a patient who suffered from SLE and had a record of travel to Southeast Asia [8].

In our case, the patient was free of disease and had no history of travelling outside of Japan. We took a detailed history, and she had no history of raw fish ingestion. There were no individuals in close association with her who developed gastroenteritis. We could not find any apparent symptoms of infection during laparoscopic surgery and hospitalization. Because she complained of severe diarrhea at admission, we assume that gastrointestinal *E. tarda* infection developed at first. Then, it probably caused development of abscess in the hematoma subsequently.

To our knowledge, there are few reports of extraintestinal *E. tarda* infection that occurred in patients without basic diseases or a history of raw fish ingestion and travelling outside of Japan.

In our case, *E. tarda* infection developed in the hematoma that originally occurred after cesarean section. It is known that a hematoma is one of the complications of a cesarean section. Some reports indicate that the frequency of hematoma is approximately 1% [9, 10]. Surgery is not always chosen for treatment of a hematoma. Some studies report that 0.3% of cesarean sections require relaparotomy to treat a hematoma [11, 12]. When there are symptoms of continuous bleeding or infection, surgery is necessary [13–15]. Otherwise, conservative therapy can be considered.

In this case, the patient's status was initially stable. Thus, we chose conservative care. On the 28th postoperative day, we found that the size of the hematoma remained unchanged, and we performed laparoscopic surgery to treat the hematoma. Some reports indicate that laparoscopic surgery is useful for treatment of a hematoma derived from cesarean section [16, 17]. Compared to laparotomy, laparoscopic surgery causes less pain and requires a shorter hospitalization period. Additionally, we can achieve a wider view and minimal invasion via laparoscopy compared to laparotomy. Surgery itself ended without short-term complications, and the patient was discharged uneventfully.

However, in this case, the hematoma was in the extrapelvic space and particularly close to the abdominal side. Therefore, it was difficult to approach the hematoma laparoscopically. We cannot deny the possibility that inadequate resection of the hematoma was the reason for the *E. tarda* infection, although surgery itself was not the reason of *E. tarda* infection. In planning surgical treatment for a hematoma, we should be flexible in consideration of the surgical procedure.

Usually, *E. tarda* is susceptible to almost all antibiotics [18, 19]. Furthermore, an extraintestinal *E. tarda* infection is known to be a critical disease. Septic shock from *E. tarda* infection is associated close to a 50% mortality rate [6]. Therefore, intensive care is necessary in the treatment of extraintestinal *E. tarda* infection. In almost all cases of an *E. tarda* abscess, antibiotic therapy is inadequate and a drainage operation needs to be performed [2, 20].

In this case, the *E. tarda* that was detected in the abscess was initially susceptible to almost all types of antibiotics, including ABPC/SBT “and TAZ/PIPC, which we administered. Because she was a noncompromised host and her fever and gastric discomfort tended to improve, we hoped that escalation of antibiotics might work more. However, the abscess enlarged, and the fever and gastric discomfort worsened rapidly. We should have performed a drainage operation, not escalation of antibiotics.

In conclusion, we experienced a case of an *E. tarda* abscess which developed in the hematoma originally derived from an emergency cesarean section. This report is the first report of an *E. tarda* abscess that developed in a patient without an underlying disease or risk factors in the postpartum period. In planning surgery to treat a hematoma, we should consider the best approach to resect and lavage the hematoma fully to prevent subsequent occurrence of an abscess. If an *E. tarda* abscess is discovered, a drainage operation should definitely be considered even if the patient is a noncompromised host, and *E. tarda* is susceptible to antibiotics.
